# Medical Society of the State of Pennsylvania

**Published:** 1858-07

**Authors:** 


MEDICAL SOCIETY OF THE STATE OF PENNSYLVANIA.—The annual meeting of this body was held at Lancaster, on the 26th of May; Dr. John L. Atlee, President, in the chair. The delegates were welcomed in a brief address by Dr. F. A. Muhlenberg. The annual address was delivered by the retiring President. Appropriate resolutions were passed in connection with the announcement of the death of Dr. Septimus A. Ogier, of Chester County, one of the Secretaries, who was killed by a collision with a railroad car, in September last. About forty delegates were present from the following counties:—Beaver, Blair, Carbon, Chester, Lancaster, Montgomery, Philadelphia, Perry, Susquehanna, and York.

Dr. R. La Roche presented a memorial from the Philadelphia Medical Society, founded in 1792, requesting a representation in the State Medical Society; the consideration of which was, after discussion, postponed until next year. An amendment to the Constitution, allowing of the formation of a new County Society for every 200,000 inhabitants, was offered by Dr. H. Carpenter, and laid over.

The Report of the Committee on Meteorology was read, containing a communication from Prof. Henry, of the Smithsonian Institute, in regard to the difficulty of obtaining correct observations upon the electrical states of the atmosphere.

On Wednesday evening, the delegates were handsomely entertained at the residences of Drs. Muhlenberg, Atlee, and Carpenter.

On Thursday, Sanitary Reports were read from the following counties:—Beaver, Berks, Blair, Carbon, Chester, Lebanon, Montgomery, Perry, Schuylkill, and Susquehanna. Biographical notices of Drs. S. A. Ogier, E. Estchell, and R. B. Dilworth, accompanied the Report from Chester County.

The Committee of Publication was directed to print the Constitution of the Society, and the Code of Ethics, with the Transactions of this year. The same Committee reported that the finances of the Society had not admitted of the publication of the form of Record for private medical statistics referred to them at the last meeting; which was therefore again referred with the same discretion.

Drs. Condie and Emerson were continued a committee to procure a supply of original vaccine virus.

The President and other officers of the Society were continued as a committee to extend the organization of the profession throughout the State.

The following gentlemen were elected officers of the Society for the ensuing year:—*President*, Dr. Smith Cunningham, of Beaver County. *Vice-Presidents*, Drs. Henry Carpenter, of Lancaster, Isaac Lefever, of Perry; William Carson, of Montgomery; and W. H. Gunkle, of Chester County. *Corresponding Secretary*, Dr. James Carson, of Philadelphia. *Recording Secretaries*, Drs. Henry Hartshorne, of Philadelphia, and John K. Raub, of Lancaster. *Treasurer*, Dr. R. P. Thomas, of Philadelphia. Besides Censors and Delegates to the American Medical Association, three gentlemen were also appointed to represent this Society in the Convention to meet at Washington in May, 1860, to revise the Pharmacopœia.

An amendment to the Constitution was unanimously adopted, altering the time of the annual session to the second Wednesday in June.

The Society adjourned at noon on Thursday, to meet at Philadelphia next year.

After the adjournment, carriages conveyed the delegates to the Franklin and Marshall College, to the Lancaster County Normal Institute, and to Wabank; where an elegant entertainment awaited them, as the guests of the Lancaster County Medical Society: a most agreeable termination to the labors of a Convention whose only subject of regret was the smallness of the number in attendance.

